# Homologous chromosome associations in domains before meiosis could facilitate chromosome recognition and pairing in wheat

**DOI:** 10.1038/s41598-022-14843-1

**Published:** 2022-06-22

**Authors:** Lorena Barea, Álvaro Redondo-Río, Rafael Lucena-Marín, Salud Serrano-Heredia, Miguel Aguilar, Pilar Prieto

**Affiliations:** 1 Plant Breeding Department, Institute for Sustainable Agriculture, Agencia Estatal Consejo Superior de Investigaciones Científicas (CSIC), Avenida Menéndez Pidal S/N., Campus Alameda del Obispo, 14004 Córdoba, Spain; 2 Área de Fisiología Vegetal, Universidad de Córdoba, Campus de Rabanales, Edif. C4, 3ª Planta, Córdoba, Spain

**Keywords:** Plant sciences, Plant breeding

## Abstract

The increasing human population demands an increase in crop yields that must be implemented through breeding programmes to ensure a more efficient and sustainable production of agro-food products. In the framework of breeding, genetic crosses are developed between cultivated species such as wheat and their relative species that are used as genetic donors to transfer desirable agronomic traits into the crop. Unfortunately, interspecific associations between chromosomes from the donor species and the cultivar are rare during meiosis, the process to produce gametes in organisms with sexual reproduction, hampering the transfer of genetic variability into wheat. In addition, little is known about how homologous (equivalent) chromosomes initiate interaction and recognition within the cell nucleus to enter meiosis. In this context, we aim to get insight into wheat chromatin structure, particularly the distribution of homologous chromosomes within the cell nucleus and their putative interactions in premeiotic stages to facilitate chromosome associations and recombination at the beginning of meiosis. Cytogenetics allows the study of both the structure and the behaviour of chromosomes during meiosis and is key in plant breeding. In this study we visualized an extra pair of barley homologous chromosomes in a wheat genetic background to study the spatial distribution, arrangements and interactions occurring exclusively between this pair of homologous chromosomes during premeiosis using fluorescence in situ hybridization (FISH). Our results suggest that homologous chromosomes can initiate interactions in premeiotic stages that could facilitate the processes of specific chromosome recognition and association occurring at the onset of meiosis.

## Introduction

Genome studies have been traditionally focused on the analysis of genetic sequences but our understanding of how they are organised in three-dimensional space has been left behind. At present, the idea of the genome as a linear sequence of nucleotides has been replaced by a dynamic three-dimensional architecture in which structural elements such as loops, domains, chromosome territories (CT) and factories are functional chromosome components regulating physical interactions within the cell nucleus^1^.

At the beginning of meiosis (the division process by which the genetic material is halved for gametes production), homologous (equivalent) chromosomes previously spread throughout the nucleus must approach and recognize each other to come into contact, recombine and, as a result, bivalents are obtained^2,3^. The way in which homologous chromosomes approach each other to interact represents one of the least understood mechanisms of the meiotic process^4–8^. Furthermore, although chromosome pairing is usually associated with meiosis, there is evidence that, at the premeiosis stage (early prophase), interactions between some areas of homologous chromosomes in interphase nuclei can occur^9^. Previous studies on chromosome pairing in wheat and its polyploid relatives have shown that chromosomes associate in pairs at the beginning of meiosis through their centromeres^3,10,11^. The fact that the first non-homologous centromere interactions occur at the beginning of meiosis or in early meiosis and before chromosome pairing, is thought to play an important role in the search for homology between chromosomes and their pairing. These homologous chromosomes must be close enough to be able to recognise the homology between the DNA sequences so that they can be paired^12^. In fact, early in the seventies, events affecting chromosome pairing were already described during premeiotic interphase before the start of leptotene^13^.

Polyploid species such as wheat are of great interest in agriculture and plant breeding, but they are also very useful for genetic studies including the examination of genome architecture and chromosome interactions during specific cellular processes, because they can tolerate both the loss of chromosome segments and complete chromosomes, as well as the addition or substitution of some chromosomes from related species. For example, wheat introgression lines carrying a pair of chromosomes of a related species are useful to study the behaviour of those chromosomes of the donor species in the wheat genetic background^14^. Thus, the studies on spatial organization of chromosome territories (the region of the nucleus occupied by a chromosome) in plants with large genomes such as wheat is allowed using GISH (genomic in situ hybridization) experiments. In situ hybridization easily enables the visualization of chromosomal introgressions from one genome into another using labelled genomic DNA from the donor species as probe. Previous studies have showed the identification of the genome of related species by chromosomal introgression lines using FISH or GISH in a wheat genetic background^15–17^. In addition to wheat, these cytogenetic studies are possible in other species^18–20^.

In a polyploid such as hexaploid (bread) wheat (*Triticum* *aestivum*, 2n = 6x = 42; **AABBDD**), which has three different subgenomes derived from three different diploid species, chromosome recognition and pairing during meiosis must be extremely regulated. Each wheat subgenome consists of seven pairs of homologous chromosomes that must recognise each other and distinguish from the equivalent chromosomes from the other subgenomes (homoeologues) to allow a diploid-like behaviour during meiosis. In most diploid organisms, meiosis includes recognition, pairing, synapsis and recombination of homologous chromosomes, which are necessary for proper and balanced segregation of bivalents. In polyploid organisms, the picture is more complicated. In the presence of more than two sets of chromosomes, the possibility of interactions between more than two related chromosomes and the formation of multivalents must be solved additional mechanisms which remained to be elucidated.

In this work, we used wheat introgression lines in which a pair of homologous chromosomes from the barley species *H.* *chilense* and *H.* *vulgare* substituted or were added to the bread wheat genetic background to study the behaviour of homologous chromosomes at the onset of meiosis. The visualization of barley homologous chromosomes in the genetic background of hexaploid wheat using in situ hybridization, allowed the study of interactions between those homologous barley chromosomes in premeiosis and the putative effect on later processes of chromosome recognition and pairing in early meiosis. Homologous chromosomes might interact in premeiosis, before leptotene (first stage of meiosis), which might contribute to facilitate chromosome recognition and association between the distal regions for homologous chromosomes where telomeres and subtelomeres are located, occurring at early meiosis. An increment in the number of premeiotic cells where homologous barley chromosomes were closer or interacting suggested that interactions between homologous chromosomes prior to meiosis do occur and could facilitate specific associations between homologues at the beginning of meiosis in wheat.

## Results

### Hordeum chilense homologous chromosome interactions, which occur in both somatic and premeiotic wheat tissues, are increased in premeiosis

**Figure 1:**
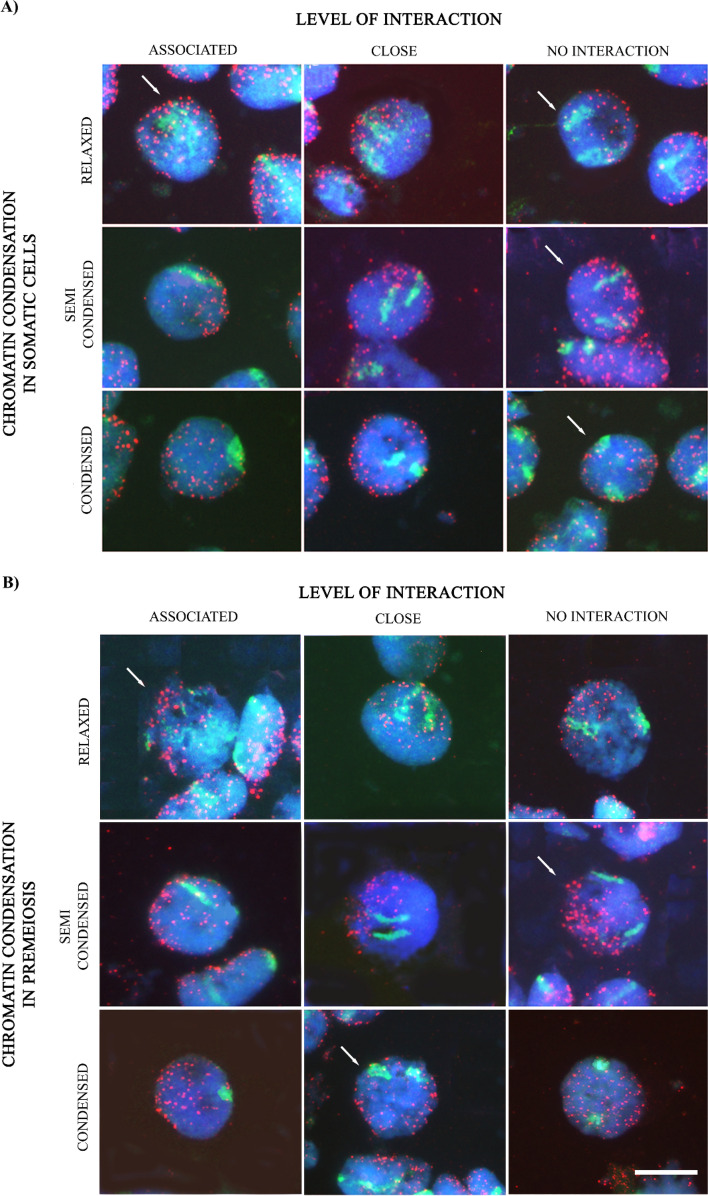
Genomic in situ hybridization to (**A**) somatic and (**B**) premeiotic chromosome spreads in wheat lines carrying 7**H**^**ch**^ *H.* *chilense* homologous chromosome substitutions. Simultaneous visualization of both *H.* *chilense* homologous (green) and telomeres (red). Columns show the spatial disposition of homologous chromosomes within the nucleus having no apparent interaction, being in proximity or associated. Rows show three different stages of chromatin condensation: relaxed, semi-condensed and condensed. Arrows were included in some panels to identify meiocytes. Scale bar represents 10 μm for all panels.

Genomic in situ hybridization has been used to visualize *H.* *chilense* homologous chromosomes in the wheat background, both in somatic and meiotic tissues, to investigate the arrangement of these wild barley chromosomes within the wheat cell nucleus. Images of the entire sample of each chromosome preparation from each wheat line were successively taken using a fluorescence microscope. At least 400 somatic and premeiotic cells (more than 1200 cells in some cases) were visualised and analysed by in situ hybridization from root tips and anthers in premeiosis for each *H.* *chilense* substitution line in hexaploid wheat, named CS(7**A**)7**H**^**ch**^, CS(7**B**)7**H**^**ch**^ and CS(7**D**)7**H**^**ch**^. Using total *H.* *chilense* genomic DNA as a probe and detected with anti-digoxigenin-FITC (green) antibody was possible to visualise only the wild barley chromosome pair in the wheat genetic background and study putative homologous interactions in premeiosis. Telomeres were also visualised to contribute to stage meiocytes in premeiosis, as telomeres at this stage are scattered around the peripheral areas of the nucleus, anchoring the chromosomes to the nuclear envelope before clustering at the telomere bouquet at the onset of meiosis. So, we were able to identify meiocytes in premeiosis both by their size, which was larger than the rest of the accompanying cells in the anther, and by the arrangement of telomeres, which are still dispersed around the nucleus (Fig. 1).

Somatic and premeiotic cells were grouped and counted according to the following criteria: (i) the arrangement and interaction of the *H.* *chilense* homologous chromosomes within the nucleus (associated, in close proximity and no-interactions/non-associated) and (ii) the degree of condensation of the DNA (condensed, semi-condensed and relaxed). *Hordeum* *chilense* introgressed chromosomes in the wheat background were interpreted as associated when the complete separation of the two chromosome structures could not be clearly discerned, targeted as close when they were in proximity within the cell nucleus but distinguishable one each, and no-interaction between them when they were at different sites of the cell nucleus (Fig. 1).

Among all the analysed nuclei, barley chromosome territories (CT) were visualized in different configurations in the wheat background nucleus for all the wheat plants analysed and, in both cases, somatic and premeiotic tissues. Both barley homologous chromosomes were clearly distinguishable from each other, and they do not appear to intermingle in a proportion of cells varying between 41.0 and 52.7% in somatic tissue and between 20.9 and 25.2% in premeiotic tissue, depending on the wheat substitution line (Table 1). However, in many cells, both barley homologous chromosomes were completely indistinguishable from each other (labelled as associated) in a proportion ranging from the 19.1 to 27.4% in somatic tissue and between 26.2 and 37.7% in premeiosis, depending on the wheat substitution line (Table 1). These results reveal that chromosome interactions between homologous chromosomes can occur in both somatic and premeiotic tissue.

**Table 1:** Total number and percentage of somatic and premeiotic cells analysed to study chromosome interactions between *H.* *chilense* homologues substituted in wheat (*T.* *aestivum* cv. Chinese spring, CS) lines.

Line	Somatic cells					Premeiosis					Associated		Close		Interaction (associated + close)	
	Associated	Close	Interaction (associated + close)	No interaction	Total	Associated	Close	Interaction (associated + close)	No interaction	Total	X^2^	"p"	X^2^	"p"	X^2^	"p"
**CS(7A)7H**^**ch**^	245^a^ (27.4%)	274^a^ (30.6%)	519^a^ (57.9%)	367 (41.0%)	895	331^a^ (37.7%)	343^a^ (39.1%)	674^a^ (76.7%)	204 (23.2%)	878	215.4	p < 0.05 (1.73 × 10^–03^)	13.9	p < 0.05 (9.37 × 10^–05^)	70.9	p < 0.05 (< 2.2 × 10^–16^)
**CS(7B)7H**^**ch**^	158^a^ (25.2%)	176^a^ (28.0%)	334 ^b^ (53.2%)	270 (42.9%)	628	106 ^b^ (26.2%)	186^a^ (46.0%)	292^a^ (72.3%)	102 (25.2%)	404	1.1	p < 0.5 (0.35)	35.0	p < 0.05 (1.62 × 10^–09^)	37.5	p < 0.05 (4.4 × 10^–10^)
**CS(7D)7H**^**ch**^	110 ^b^ (19.1%)	156^a^ (27.1%)	266 ^c^ (46.3%)	303 (52.7%)	575	318 ^b^ (30.9%)	479^a^ (46.5%)	797^a^ (77.4%)	216 (20.9%)	1030	26.0	p < 0.05 (1.69 × 10^–04^)	57.9	p < 0.05 (1.35 × 10^–14^)	159.8	p < 0.05 (< 2.2 × 10^–16^)

The significance (p value) of the data between somatic and premeiotic cells at the same level of chromosome interaction within the same wheat line was confirmed by Student’s t test. The robustness of the data was indicated by the X^2^. Data with the same letter within a column represent no significantly differences (when p < 0.05) among wheat lines at the same level of chromosome interaction.

Barley homologous chromosomes were also visualized occupying proximal regions within the wheat nucleus, although they were readily distinguishable from each other, in a proportion that ranged from 27.1 to 30.6% in somatic tissue and between 39.1 and 46.5% in premeiosis, depending on the wheat substitution line (Table 1). Homologous chromosomes located in proximity within the wheat nucleus might also suggest some molecular interactions between them.

With the aim of studying whether chromosome interactions between homologous chromosomes were promoted in premeiosis in wheat, since this kind of interactions prior to meiosis could facilitate recognition and association between homologues at later stages, the position of *H.* *chilense* homologous chromosomes in premeiotic tissues was compared with the observations made in somatic cells by in situ hybridization in the CS(7**A**)7**H**^**ch**^, CS(7**B**)7**H**^**ch**^ and CS(7**D**)7**H**^**ch**^ wheat substitution lines. The use of these three wheat lines also allowed the analysis of differences that could be associated to the specific wheat subgenome whose chromosome pair was substituted by the *H.* *chilense* one. Thus, chromosome preparations of roots and anthers in premeiosis from *H.* *chilense* substitutions in wheat were exhaustively examined by GISH to compare statistically the degree of interactions of homologous chromosomes in premeiosis respecting the somatic tissue.

A significant increase in the number of cells in premeiosis showing associated *H.* *chilense* homologous chromosomes was observed compared with the number of somatic cells where alien barley chromosomes were also associated (Table 1). The proportion of cells in premeiosis displaying associated homologous barley chromosomes, which ranged from 26.2 to 37.7%, depending on the wheat line analysed, was higher than the percentage of somatic cells showing associated homologous chromosomes, whichvaried between 19.1 and 27.4%. Thus, on average, we found an increase of almost 10% of cells in premeiosis with respect to somatic cells having associated homologous barley chromosomes. This increase in the number of cells in premeiosis compared with somatic cells had statistical significance for all the wheat line analysed (p < 0.05 for CS(7A)7H^ch^ and CS(7D)7H^ch^ wheat lines and p < 0.5 for CS(7B)7H^ch^ wheat line) (Table 1). These results suggest that putative interactions between barley homologues are promoted in stages prior to meiosis in the CS(7**A**)7**H**^**ch**^, CS(7**B**)7**H**^**ch**^ and CS(7**D**)7**H**^**ch**^ wheat substitution lines.

Differences in the number of cells displaying associated barley homologous chromosomes were found depending on which wheat chromosome was substituted by *H.* *chilense* chromosome 7**H**^**ch**^ both in somatic and premeiotic tissues (indicated with different letters within a column in Table 1).

In interphase nuclei, homologous chromosomes not only interact directly between them but also other interactions through nuclear structures, proteins or more complex assemblies can occur. Thus, once we observed an increment on the number of premeiotic cells displaying both barley homologous chromosomes physically associated, we also analysed the number of cells in premeiosis in which both homologues were in close proximity within the wheat nucleus, based on the hypothesis that secondary interactions between homologous chromosomes might occur when they are in proximity although physical interactions were not cytological appreciated. These results were compared with the number of cells in somatic tissues with equivalent disposition of the barley chromosomes. The number of premeiotic cells showing the two barley chromosomes in proximity, which varied between 39.1 and 46.5% depending on the substitution line analysed, was higher than the equivalent disposition of the barley chromosomes in somatic tissue, which varied between 27.1 and 30.6%, depending on the wheat line (Table 1). These observations represented an increment in the number of premeiotic cells displaying both homologous chromosomes in proximity within the cell nucleus of approximately 15%, which was also statistically significant (p < 0.05 for the three wheat substitution lines (Table 1).

No statistical differences related to the wheat subgenome substituted by the *H.* *chilense* chromosome were found in premeiosis when homologous barley chromosomes were in proximity in the nucleus (indicated with the same letter within a column for CS(7A)7H^ch^, CS(7B)7H^ch^ and CS(7D)7H^ch^ wheat lines (p < 0.05).

We also performed the comparisons and the statistical analysis on the number of cells displaying both associated homologous chromosomes and chromosomes in close proximity, based on the hypothesis that chromosome interactions can occur in both situations. We observed a percentage ranging between 72.3 and 77.6% (depending on the wheat line analysed) in premeiosis compare to 46.3% to 57.9% in somatic tissue, which means more than 20% increase in homologous chromosome interactions in premeiosis in comparison to somatic cells (Table 1). This increase in the number of cells showing wild barley homologous chromosomes interacting at premeiosis stages was statistically significant for the three wheat lines studied (p < 0.05 in all the cases), suggesting that cytogenetic and molecular interactions between homologous chromosomes might be promoted in early meiosis in wheat and could facilitate later chromosome associations in meiosis.

In addition, , no significant differences in the number of cells displaying interactions in premeiosis between wild barley homologous chromosomes were found among the different *H.* *chilense* substitution lines in wheat (same letter within a column for CS(7A)7H^ch^, CS(7B)7H^ch^ and CS(7D)7H^ch^ wheat lines (p < 0.05) (Table 1), suggesting that the degree of interaction between homologous chromosomes in premeiosis is independent of the wheat subgenome whose chromosome has been substituted. Thus, our results mean that genetic factors involved in homologous chromosome interactions are conserved among the different subgenomes.

### Homologous chromosome interactions are promoted in premeiosis in wheat independently of the introgressed genome

**Figure 2:**
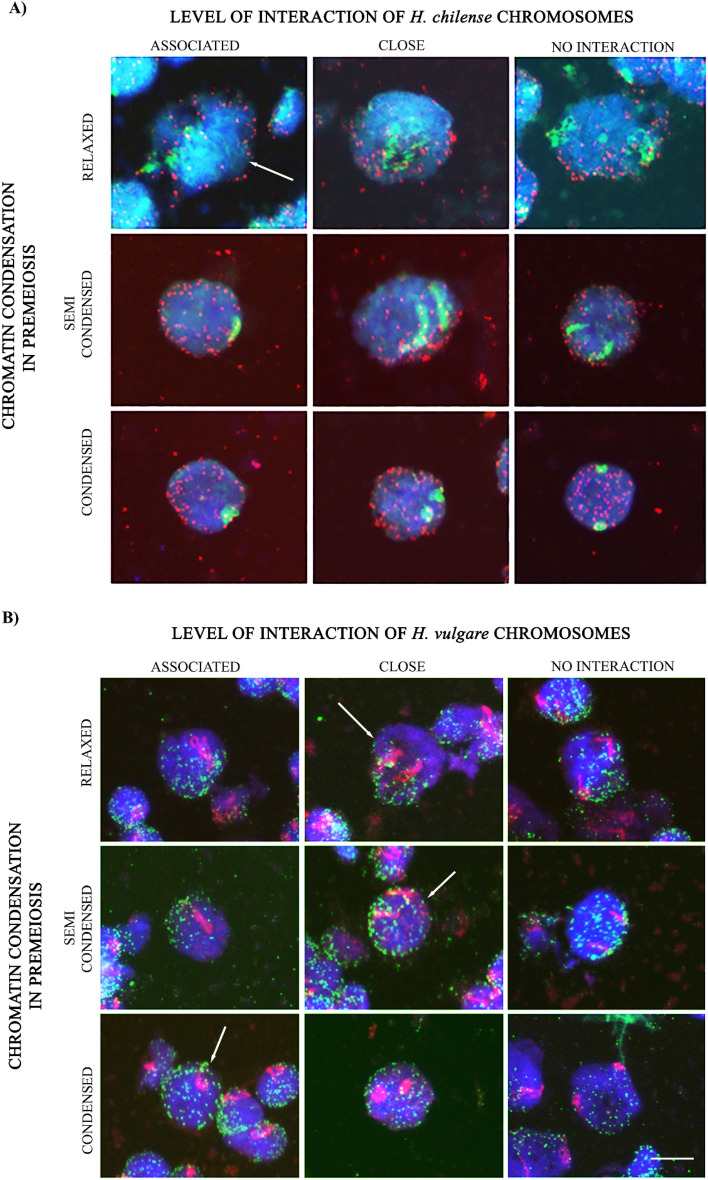
Genomic in situ hybridization to premeiotic chromosome spreads in wheat lines carrying 7**H**^**ch**^ *H.* *chilense* and 7**H**^**v**^ *H.* *vulgare* additions, respectively. Simultaneous visualization of (**A**) *H.* *chilense* chromosomes (green) and telomeres (red) and (**B**) *H.* *vulgare* homologous chromosomes (red) and telomeres (green). Columns show the spatial disposition of homologous chromosomes within the nucleus having no apparent interaction, being in proximity or associated. Rows show three different stages of chromatin condensation in somatic cells: relaxed, semi-condensed and condensed. Scale bar represents 10 μm for all panels.

Considering that homologous chromosomes associations were promoted in premeiotic stages in wheat lines carrying *H.* *chilense* chromosome substitutions, we focused on studying whether homologous chromosome associations were also promoted in premeiosis when the *H.* *chilense* homologous chromosomes were added to the whole wheat genome. In addition, we also tested homologous chromosome associations in premeiosis when another genome, such as *H.* *vulgare*, was added to wheat. Thus, we analysed homologous chromosome associations in premeiosis in *H.* *chilense* and *H.* *vulgare* chromosome 7 addition lines in wheat. Chromosome spreads from somatic and premeiotic tissues were carefully screened by in situ hybridization as performed previously for the *H.* *chilense* substitution lines. We scored homologous chromosomes associated, proximal or without apparent interactions to assess whether a promotion of homologous chromosome associations occur in premeiosis (Fig. 2). More than 800 cells were counted from each *H.* *chilense* and *H.* *vulgare* chromosome 7 addition lines in wheat in both somatic and premeiotic tissues (Table 2). The number of cells displaying the wild and cultivated barley homologous chromosomes associated, proximal and non-interacting in premeiosis were compared with equivalent chromosome patterns in somatic cells (Fig. 2 and Supplementary Fig. 1). Results showed that, although there are homologous chromosome associations in somatic tissues for both *H.* *chilense* and *H.* *vulgare* additions (25.7% and 27.0%, respectively), the number of cells in premeiosis displaying both *H.* *chilense* and *H.* *vulgare* homologous chromosomes associated was higher (33.7% and 34.3%, respectively, p < 0.05 in both addition lines) (Table 2).

**Table 2:** Total number and percentage of somatic and premeiotic cells analysed to study chromosome interactions between *H.* *chilense* and *H.* *vulgare* homologous chromosomes added to wheat (*T.* *aestivum* cv. chinese spring, CS) lines.

Line	Somatic cells					Premeiosis					Associated		Close		Interaction (associated + close)	
	Associated	Close	Interaction (associated + close)	No interaction	Total	Associated	Close	Interaction (associated + close)	No interaction	Total	X^2^	"p"	X^2^	"p"	X^2^	"p"
**CSA7H**^**ch**^	318^a^ (25.7%)	410^a^ (33.2%)	728^a^ (58.9%)	507 (41.0%)	1235	280^a^ (33.7%)	231^a^ (27.8%)	511^a^ (61.5%)	320 (38.5%)	831	15.2	p < 0.05 (4.71 × 10^–05^)	6.8	p > 0.5 (0.99)	1.3	p < 0.5 (0.12)
**CSA7H**^**v**^	262^a^ (27.0%)	380^a^ (39.2%)	642^a^ (66.2%)	328 (33.8%)	970	443^a^ (34.3%)	561^a^ (43.4%)	1004^a^ (77.6%)	289 (22.3%)	1293	13.6	p < 0.05 (1.14 × 10^–04^)	4.0	p < 0.05 (0.022)	36.7	p < 0.05 (6.80 × 10^–10^)

The significance (p value) of the data between somatic and premeiotic cells at the same level of chromosome interaction within the same wheat line was confirmed by Student’s t test. The robustness of the data was indicated by the X^2^. Data with the same letter within a column represent no significantly differences (when p < 0.05) among both wheat addition lines at the same level of chromosome interaction.

No statistical differences were found between *H.* *chilense* or *H.* *vulgare* chromosome additions either in somatic and premeiotic tissues showing associated barley chromosomes (same letter with a column for both somatic and premeiotic tissues for CSA7H^ch^ and CSA7H^v^ wheat lines, p < 0.05) (Table 2), suggesting that homologous chromosome associations occur in somatic tissue and were promoted in premeiosis in wheat independently of the homologous chromosome pair studied.

Similarly, we visualised *H.* *chilense* and *H.* *vulgare* homologous chromosomes in proximal locations within the cell nucleus in both somatic and premeiotic tissues. An increase in the number of cells in premeiosis showing both *H.* *chilense* and *H.* *vulgare* homologous chromosomes interacting (61.5% and 77.6%, respectively) was observed compared to an equivalent homologous chromosome configuration in somatic tissues (58.9% and 66.2%, respectively; Table 2). No differences between CSA7H^ch^ and CSA7H^v^ wheat lines were found for the level of chromosome configuration (proximal; same letter within the column, p < 0.05). Our results suggest that homologous chromosome interactions in premeiosis are promoted in the wheat background and these chromosome interactions are not chromosome or genome specific.

## Discussion

Chromosomes experienced diverse changes around the cell cycle. These changes affect both their local and global architecture and their interactions with nuclear structures including other chromosomes. All these changes are particularly important in the context of cell division (mitosis and meiosis) among other processes such as regulation of gene expression, cell development and response to environmental changes and/or stress conditions. A few questions around chromosome architecture and dynamics remain unsolved. For example, how chromosomes are organised in the nucleus and how they interact each other in key processes occurring in early meiosis are still unclear. This includes the homologous pairing enigma, that is how chromosomes interact to recognise each other and associate correctly in pairs and whether chromosome associations are promoted before meiosis (premeiotic stages) to facilitate such homologous chromosome associations. These processes need to be elucidated, since they are key in the framework of breeding. This knowledge will facilitate the transfer of existing genetic variability in crop-related species such as wild and cultivated barley species (*H.* *chilense* and *H.* *vulgare*, respectively) into an important crop species like wheat. The lack (or low level) of interspecific chromosome pairing impedes recombination between the chromosomes from wheat and the added or substituted chromosomes from related species, hence it limits the transfer of desirable traits from donor species into wheat. The importance of plant breeding has contributed to put the spotlight on the study of chromosome dynamics and chromosome interactions occurring during meiosis^21^.

In this work we go deeper into the knowledge of chromosome interactions between homologues in premeiosis in the wheat genetic background, since these premeiotic interactions might facilitate chromosome recognition and pairing during early meiosis. Using *H.* *chilense* and *H.* *vulgare* chromosome introgressions in wheat lines, we have visualized one pair of homologous chromosomes in the wheat background both in somatic and premeiotic cells using GISH as performed previously^22^. In plants, there is evidence of constitutive homologous chromosome pairing. A study in *Brachypodium* *distachyon* root cells interphase nuclei showed that the association of homologous chromosomes is more frequent than expected in a random arrangement of all chromosomes within the nucleus^23^. The study of polyploid organisms like wheat and *Brassica* *napus* has shown that chromosomes of the different subgenomes are not intermingled but segregated, so that all chromosomes of a subgenome occupy a kind of genome territory, being the interactions among chromosomes of the same subgenome more probable and intense^24^. In the case of bread wheat, its genome includes three subgenomes (A, B, D) that would also occupy three different genome territories within the nucleus^25^. Besides our results with *Hordeum* introgressions, similar experiments in wheat somatic tissue carrying rye genetic introgressions concluded that a fraction of somatic cells (around 15%) showed interaction between homologous chromosomes^19^. In our case, we found a 27% of somatic cells in which homologous chromosomes appeared to be associated. However, in comparison with somatic cells, we found an increase in the number of premeiotic cells in which both *H.* *chilense* and *H.* *vulgare* pairs of homologous chromosomes were associated, suggesting a promotion of homologous chromosome interactions in premeiosis in wheat. No significant differences on the number of cells displaying interacting homologous chromosomes were observed in premeiosis among the different *H.* *chilense* substitution lines, what suggests that the process of interaction between homologues in the wheat genetic background is not determined by any specific subgenome. Equivalent results were also obtained when *H.* *chilense* or *H.* *vulgare* chromosomes were added to the whole wheat genome. Taken together, our results suggest that premeiotic homologous pairing is promoted in wheat, might facilitate chromosome recognition and pairing at the onset of meiosis and genetic factors involved in homologous chromosome interactions might be also conserved.

In situ hybridization in chromosome spreads was already used several decades ago to show somatic and premeiotic homologous pairing by multiple transient interstitial interaction in yeast^26,27^. Recently, Takada et al.^28^ showed that DNA methylation in mammal premeiotic germ cells seems to regulate meiotic prophase by facilitating homologous chromosome pairing. No premeiotic chromosome associations have been described in diploid plants such as *Arabidopsis*, maize and diploid progenitors of wheat^18,29^. In contrast, several studies on chromosome pairing between hexaploid wheat, tetraploid wheat and related polyploid species have demonstrated the association of chromosomes in pairs via their centromeres before the onset of meiosis during anther development^3,11^. Premeiotic associations between homologous rye chromosome arms have been shown in wheat-rye lines using in situ hybridization^30,31^. A study in autotetraploid *Arabidopsis* suggests that polyploidization reduces intra-arm interactions and increases inter-chromosome interactions^32^. Premeiotic chromosome pairing through centromeres and telomeres has also been observed in rice^33^.

The observed interactions between homologous chromosomes both in interphase nuclei and in premiotic cells could be explained just by Brownian motion within the nucleus. However, there are multiple factors that should be considered. The existence of specific interactions between chromosomes and other structures, together with sequence and architecture similarities between homologues, suggests that homologous chromosomes would tend to occupy nearby regions. In general, the great similarity between homologous chromosomes suggests that any interaction with other structures or participation in any process will tend to pair homologues instead of homeologues or heterologous chromosomes. The processes that would facilitate inter-homologue interactions include transcription at transcription factories, trans-regulation of gene expression, replication and DNA repair.

Transcription seems to be particularly relevant, since it could initiate somatic pairing of homologous chromosomes^34^. Homologous chromosomes, having identical chromosome architecture, also display almost identical patterns of transcription factories and heterochromatin, and they are thought to be joined at the transcriptional factories^35^. Non-coding RNAs accumulate on their gene loci, and they could contribute to the association of homologous chromosomes through allelic loci^36^. Inter-chromosomal contacts have also been related to gene regulation in trans. Lancôt et al.^37^ suggested that gene expression could be repressed by direct interactions between two copies of a gene placed on different chromosomes. Multiple examples of gene regulation by inter-chromosomal contacts have been described in animals^38^.

Replication seems to imply inter-chromosomal interactions. In fission yeast, they found evidence for a mechanism of site-specific replication termination stimulated by inter-chromosomal interactions between replication termination sites^39^. In *Arabidopsis*, they found a correlation between chromosomal interactions and genomic regions that replicate during the interphase, suggesting that nearby sequences tend to replicate at the same time^40^. A similar picture was found in a study involving time and position parameters of DNA replication in several *Poaceae* including wheat^41^. Homologous inter-chromosomal interactions seem to be facilitated by DNA repair, a process that is extremely important in plants, due to their intense exposure to heavy metal, ionizing radiation, and other biotic and biotic sources of stress, on top of endogenous processes that could damage DNA. Double strand break (DSB) is the most severe DNA damage. Among other mechanisms, DNA repair can proceed by homologous recombination (HR) via synthesis dependent strand annealing (SDSA) between homologous chromosomes^42^. In tomato, induced allele dependent DSB repair was proposed^42,43^. Filler et al.^44^ showed that the induction of DSBs in tomato somatic cells via CRISPR-Cas9 increases the frequency of homologous contact and recombination between homologous chromosomes, demonstrating that the meiotic HR machinery is not necessary for DSB-induced homologues recombination^45^.

The fact that multiple processes like transcription, genetic regulation, replication and DNA repair allow inter-chromosomal contacts throughout the whole cell-cycle points to the relevance of all these processes to explain homologous chromosome pairing and recombination in the interphase, in somatic cells, as well as in reproductive cells and meiosis. As already pointed out^46^, our observations in wheat suggest that chromosome interactions between homologous chromosomes are initiated before meiosis and could contribute to promote proper pairing of homologues before chromosome association through the axis and synaptonemal complex formation in meiosis. These findings also support the hypothesis that there must be a feature of the genomic architecture that might facilitate chromosome movements before the onset of meiosis to allow homologous chromosomes to be in proximity and interact to assist homologous recognition and pairing independently of recombination and DNA damage repair later in meiosis. This hypothesis is supported by the increase of the fraction of premeiotic cells displaying associated or proximal homologous chromosomes within the cell nucleus shown in this work.

Although some genes like *HOP1*, *REC8* and *RED1* in yeast, and the homologues in plants ASY1/PAIR2, REC8-like and ASY3/PAIR3, respectively, have been proposed to play key roles in chromosome associations, the initial interactions between homologous chromosomes to recognize each other before an efficient association in pairs, and the molecular factors involved, are still unclear^47–55^. Some members of the HMG family of proteins, particularly the HMGA subfamily, could also play an important function in homologous chromosome pairing through their putative interaction at AT-rich sites accessible on the protruding regions of DNA loops^56,57^. Further studies will be needed to test the hypothesis of a mechanism in which homologous chromosomes could interact via DNA protruding loops and pairing proteins that might stabilize the associations between homologous chromosomes in proximal locations within the nucleus in premeiosis and facilitate specific homologous chromosome pairing at the onset of meiosis in wheat.

## Methods

### Plant material

In this work, we used *Hordeum* *chilense* chromosome 7**H**^**ch**^ substitutions in bread wheat (*Triticum* *aestivum* cv. Chinese Spring) lines in which the *H.* *chilense* chromosome 7**H**^**ch**^ pair replaced its homoeologous pair from the wheat **A**, **B** and **D** genomes, respectively. In detail, *H.* *chilense* substitutions lines in wheat were: CS(7**A**)7**H**^**ch**^ (6 pairs **AA** + 7 pairs **BB** + 7 pairs **DD** + pair 7**H**^**ch**^), CS(7**B**)7**H**^**ch**^ (7 pairs **AA** + 6 pairs **BB** + 7 pairs **DD** + pair 7**H**^**ch**^) and CS(7**D**)7**H**^**ch**^ (7 pairs **AA** + 7 pairs **BB** + 6 pairs **DD** + pair 7**H**^**ch**^). We have also used bread wheat lines (*T.* *aestivum* cv. Chinese Spring) carrying *H.* *chilense* and *H.* *vulgare* additions for chromosomes 7**H**^**ch**^ and 7**H**^**v**^, respectively. Thus, these addition lines carried the full genome of hexaploid wheat (**AABBDD**) plus the pair 7**H**^**ch**^ and 7**H**^**v**^, respectively. All the wheat lines have been provided by Dr. Steve Reader from the John Innes Centre (Norwich, UK). We have focused our study on homologous chromosome interactions in chromosome 7**H**^**ch**^ because substitution lines of *H.* *chilense* chromosome 7**H**^**ch**^ for each wheat **A**, **B** and **D** homoeologous chromosome pairs are available, additionally to the 7**H**^**ch**^ and 7**H**^**v**^ wheat addition lines.

### Somatic cells analysis

Somatic chromosome spreads were prepared from root tip cells. Seeds were germinated on wet filter paper at 25 °C in the dark. Root tips from germinating seeds were cut after 24 h incubation, and pre-treated with colchicine (0.05%, w/v) during 4 h at 25 °C in the dark. Root tips were then fixed in a freshly prepared ethanol–acetic acid mix (3:1 v/v) and stored at 4 °C for at least 1 month. Plants were grown in a growth chamber under controlled temperature (26 °C during the day and 18 °C during the night, 16 h photoperiod).

Genomic DNA from *H.* *chilense* *and* *H.* *vulgare* was used to identify and visualize the pair or *H.* *chilense* and *H.* *vulgare* chromosomes, respectively, in somatic cells in the wheat background. The in situ hybridization protocol was performed as described previously^22^. Briefly, total genomic DNA from *H.* *chilense* and *H.* *vulgare* was labelled by nick translation with digoxigenin-11-dUTP (Roche Applied Science, Indianapolis, IN, USA) and biotin-11-dUTP (Boehringer Mannheim Biochemicals, Germany), respectively, to be used as probes for in situ hybridization experiments in somatic cells. The final concentration of each probe was 5 ng/μL in the hybridization mix (50% formamide, 2 × SCC, 5 ng of each digoxigenin and biotin-labelled probes, 10% dextran sulphate, 0.14 μg of yeast tRNA, 0.1 μg of sonicated salmon sperm DNA, and 5 ng of glycogen. Posthybridization washes were conducted twice at 2 × SSC (5 min each) at 37 °C plus one extra wash in 1 × SSC at room temperature (RT). Biotin- and digoxigenin-labelled probes were detected with streptavidin-Cy3 conjugates (Sigma, St. Louis, MO, USA) and antidigoxigenin FITC antibodies (Roche Diagnostics, Meylan, France), respectively. Total DNA was counterstained with 4′,6-diamidino-2- phenylindole (DAPI) and mounted in Vectashield (Vector Laboratories, Burlingame, CA, USA). Hybridization results were visualized using a Nikon Eclipse 80i epifluorescence microscope and images were captured with a Nikon CCD camera using the Nikon 3.0 software (Nikon Instruments Europe BV, Amstelveen, The Netherlands) and processed with Photoshop 11.0.2 software for adjustment of brightness and contrast (Adobe Systems Inc., San Jose, CA, USA).

### Premeiotic cells analysis

Mature plants were used to collect spikes in premeiosis, which were conserved in 100% ethanol–acetic acid 3:1 (*v*/*v*) until they were used to study homologous chromosome interactions. Pollen mother cells (PMCs) at premeiosis were used to prepare chromosome spreads. A drop of 45% glacial acetic acid was applied to macerate anthers on ethanol-cleaned slides, which were squashed under a cover slip, and dipped in liquid nitrogen to fix the plant material on the slide. The cover slip was removed, and the slides were air-dried and stored at 4 °C until used.

Total genomic DNA from *H.* *chilense* *and* *H.* *vulgare* was labelled by nick translation with digoxigenin-11-dUTP (Roche Applied Science, Indianapolis, IN, USA) and biotin-11-dUTP (Boehringer Mannheim Biochemicals, Germany), respectively, to be used as probes for in situ hybridization experiments in PMCs. The conserved telomeric sequence from *A.* *thaliana* (AAATCCC)^58^ was also labelled by nick translation with digoxigenin-11-dUTP or biotin-11-dUTP, indistinctly, to allow the visualization of the telomeres from all chromosomes, indistinctly, and contribute to stage the PMCs in premeiosis.

GISH analysis allowed the visualization of the *H.* *chilense* *and* *H.* *vulgare* chromosomes and their interactions during premeiosis in the wheat background, as described previously for somatic cells^22^. As in somatic cells analysis, the final concentration of each probe was 5 ng/μL in the hybridization mix (50% formamide, 2 × SCC, 5 ng of each digoxigenin and biotin-labelled probes, 10% dextran sulphate, 0.14 μg of yeast tRNA, 0.1 μg of sonicated salmon sperm DNA, and 5 ng of glycogen). Posthybridization washes were equivalent to the ones performed for somatic cells analysis. Biotin-labelled and digoxigenin-labelled probes were detected with a streptavidin-Cy3 conjugate and antidigoxigenin-FITC, respectively. Chromosomes were counterstained with DAPI (4′, 6-diamidino-2-phenylindole) and mounted in Vectashield (Vector Laboratories, Burlingame, CA, USA). Hybridization results were visualized as previously described for somatic samples.

### Statistical analysis

Statistical analysis of the data was performed by applying a t-Student distribution analysis using the RStudio v/1.1.456 program available at https://www.rstudio.com/products/rstudio/. Results were considered statistically significant when p ≤ 0.05. More than 15 plants from each wheat line were used to prepare at least 3 chromosome spreads both in somatic and premeiosis from each plant. A minimum of 400 cells were analysed using in situ hybridization both in somatic and premeiosis stages in each wheat line to obtain statistically robust results.

## Supplementary Information

Supplementary Figure 1.

## Data Availability

All data generated or analysed during this study are included in this published article [and its supplementary information files].
