# Supplementary material for: Homologous chromosome associations in domains before meiosis could facilitate chromosome recognition and pairing in wheat
Source: Sci Rep. 2022 Jun 22;12:10597. doi: 10.1038/s41598-022-14843-1 (PMC9217977; doi:10.1038/s41598-022-14843-1)

**Supplementary information.**

Supplementary Figure 1. Genomic *in situ* hybridization to somatic chromosome spreads in wheat lines carrying 7**H^ch^** *H. chilense* and 7**H^v^** *H. vulgare* additions, respectively. Visualization of *H. chilense* (green) and *H. vulgare* (red) homologous chromosomes. Columns show the spatial distribution of homologous chromosomes within the nucleus having no apparent interaction, being in proximity or associated. Rows show three different stages of chromatin condensation in somatic cells: relaxed, semi-condensated and condensated. Scale bar represents 10 μm for all panels.


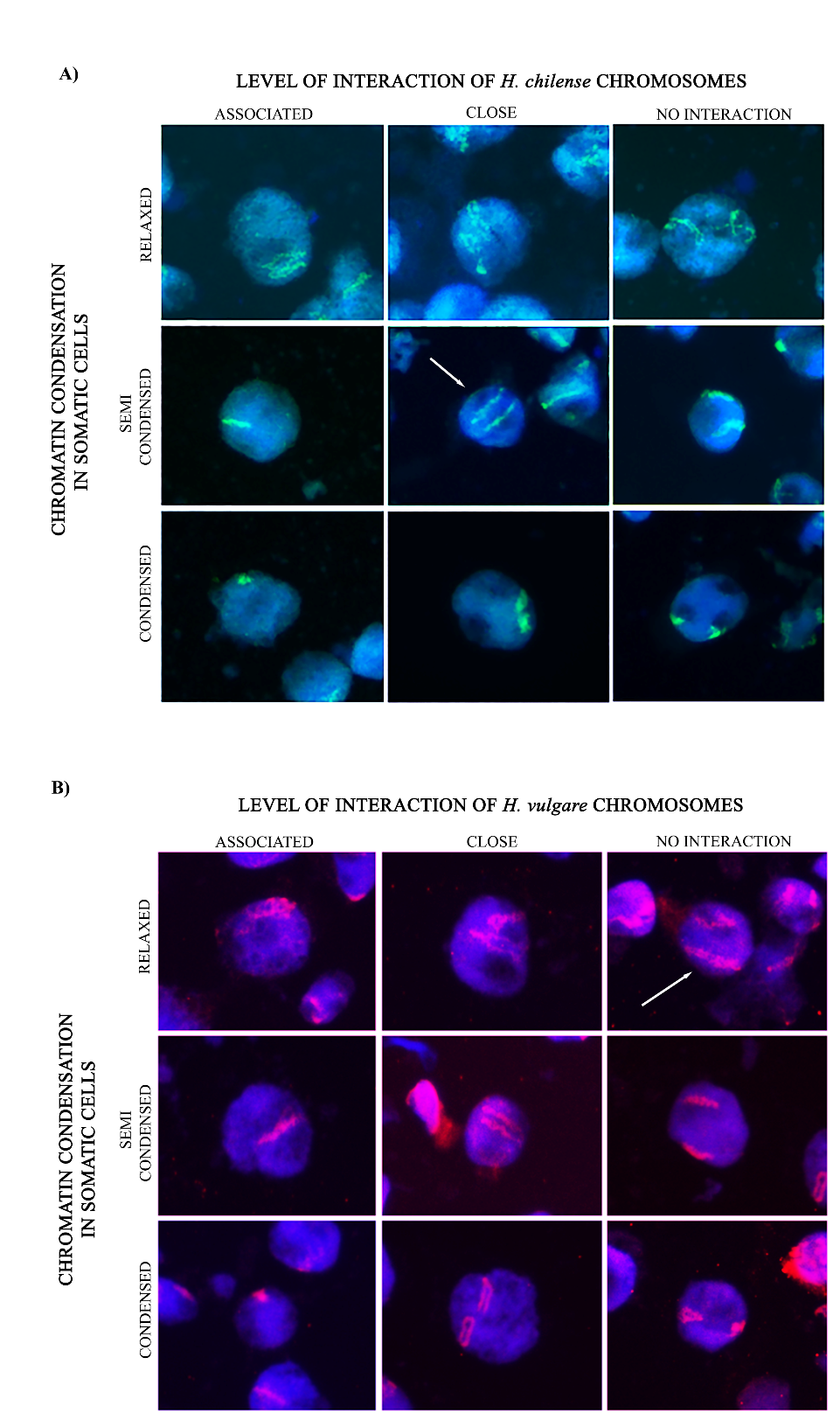

Supplement: Supplementary file 1 — Supplementary Figure 1. [file 41598_2022_14843_MOESM1_ESM.docx]
